# Proliferation and Differentiation of *Trypanosoma cruzi* inside Its Vector Have a New Trigger: Redox Status

**DOI:** 10.1371/journal.pone.0116712

**Published:** 2015-02-11

**Authors:** Natália P. Nogueira, Francis M. S. Saraiva, Pedro E. Sultano, Paula R. B. B. Cunha, Gustavo A. T. Laranja, Graça A. Justo, Kátia C. C. Sabino, Marsen G. P. Coelho, Ana Rossini, Georgia C. Atella, Marcia C. Paes

**Affiliations:** 1 Laboratório de Interação Tripanossomatídeos e Vetores—Departamento de Bioquímica, Instituto de Biologia Roberto Alcantara Gomes (IBRAG), Universidade do Estado do Rio de Janeiro (UERJ), Rio de Janeiro, Brasil; 2 Instituto Nacional de Ciência e Tecnologia—Entomologia Molecular (INCT-EM)—Rio de Janeiro, Brasil; 3 Laboratório de Bioquímica de Lipídeos e Lipoproteínas-Instituto de Bioquímica Médica Leopoldo de Meis (IBqM), Universidade Federal do Rio de Janeiro (UFRJ)—Rio de Janeiro, Brasil; 4 Laboratório de Imunologia Aplicada à Bioquímica de Proteínas e Produtos Naturais—Departamento de Bioquímica, Instituto de Biologia Roberto Alcantara Gomes (IBRAG), Universidade do Estado do Rio de Janeiro (UERJ), Rio de Janeiro, Brasil; 5 Laboratório de Toxicologia e Biologia Molecular, Departamento de Bioquímica, Instituto de Biologia Roberto Alcantara Gomes (IBRAG), Universidade do Estado do Rio de Janeiro (UERJ), Rio de Janeiro, Brasil; George Washington University School of Medicine and Health Sciences, UNITED STATES

## Abstract

*Trypanosoma cruzi* proliferate and differentiate inside different compartments of triatomines gut that is the first environment encountered by *T. cruzi*. Due to its complex life cycle, the parasite is constantly exposed to reactive oxygen species (ROS). We tested the influence of the pro-oxidant molecules H_2_O_2_ and the superoxide generator, Paraquat, as well as, metabolism products of the vector, with distinct redox status, in the proliferation and metacyclogenesis. These molecules are heme, hemozoin and urate. We also tested the antioxidants NAC and GSH. Heme induced the proliferation of epimastigotes and impaired the metacyclogenesis. β-hematin, did not affect epimastigote proliferation but decreased parasite differentiation. Conversely, we show that urate, GSH and NAC dramatically impaired epimastigote proliferation and during metacyclogenesis, NAC and urate induced a significant increment of trypomastigotes and decreased the percentage of epimastigotes. We also quantified the parasite loads in the anterior and posterior midguts and in the rectum of the vector by qPCR. The treatment with the antioxidants increased the parasite loads in all midgut sections analyzed. *In vivo*, the group of vectors fed with reduced molecules showed an increment of trypomastigotes and decreased epimastigotes when analyzed by differential counting. Heme stimulated proliferation by increasing the cell number in the S and G2/M phases, whereas NAC arrested epimastigotes in G1 phase. NAC greatly increased the percentage of trypomastigotes. Taken together, these data show a shift in the triatomine gut microenvironment caused by the redox status may also influence *T. cruzi* biology inside the vector. In this scenario, oxidants act to turn on epimastigote proliferation while antioxidants seem to switch the cycle towards metacyclogenesis. This is a new insight that defines a key role for redox metabolism in governing the parasitic life cycle.

## Introduction

The parasitic protozoan *Trypanosoma cruzi*, which is responsible for Chagas disease [1], presents four distinct stages in its complex life cycle. During the blood meal in an infected mammalian host, the insect vector ingests the bloodstream form of *T*. *cruzi*, which initiates the development of the parasite in the intestinal lumen of bloodsucking triatomine bugs. A few hours after the meal, in the anterior region of the midgut, bloodstream trypomastigotes transform into proliferative, non-infective epimastigotes. In the rectum of the vector, a new differentiation (metacyclogenesis) takes place, where epimastigotes transform into non-proliferative, infective metacyclic trypomastigotes. These metacyclic trypomastigotes are then released along with the feces and urine of the insect and may reach the bloodstream of a new vertebrate host, where they infect mainly macrophages or cardiac and smooth muscle fibers. In these cells, the parasite undergoes another dramatic transformation into proliferative intracellular amastigotes. After intense multiplication inside the host cell, the amastigotes become bloodstream trypomastigotes that can infect other host cells or reach the circulatory system, completing the cycle [2, 3].

Since its discovery over a century ago [1], evidence in the literature has indicated that the association between *T*. *cruzi* and triatomines is essential for successful Chagas disease propagation [4]; thus, several factors and molecules were shown to be important to establishing the infection. However, many aspects of these complex interactions remain to be elucidated.

The first environment encountered by *T*. *cruzi* after the blood meal is the midgut of the insect, where large amounts of hemoglobin (Hb) are degraded resulting in the release of huge concentrations of heme, a molecule known to increase the formation of reactive oxygen species (ROS) and alter membrane selectivity and permeability [5, 6]. Evidence in the literature has indicated that blood-feeding insects manage heme toxicity using several adaptations to ameliorate or prevent heme-induced damage. *Rhodnius*. *prolixus*, a Chagas disease vector, uses heme aggregation to form hemozoin as an efficient detoxification pathway [7, 8].

Thus, the anterior region of the midgut represents a potentially oxidative environment, rich in nutrients, and it is also where epimastigotes proliferate intensely. In fact, our group has demonstrated that heme induces *T*. *cruzi* epimastigote proliferation *in vitro* in a dose-dependent manner [9] and that this heme-induced *T*. *cruzi* growth is associated with calcium-calmodulin-dependent kinase II (CaMKII) activity [10]. We also recently showed that heme induces a transient oxidative stress condition that stimulates *T*. *cruzi* growth via a mechanism mediated by the CaMKII pathway [11].

Additionally, the transformation of epimastigotes into metacyclic trypomastigotes (metacyclogenesis), a process mandatory to the completion of the *T*. *cruzi* life cycle, occurs in the final compartment of the intestinal tract (the posterior region of the small intestine and the rectum) [12]. Over the years, several factors have been implicated to influence metacyclogenesis, such as the strain or clone used [13, 14], osmolarity [15, 16], the initial pH of the media [17], the use of L-proline as the only source of carbon and nutritional stress [18, 19]. However, the molecular bases of the morphogenetic alterations necessary and sufficient to elicit parasite differentiation remain to be fully elucidated. Recently, Tonelli et al., 2011 [20], shed a light into the problem demonstrating that nutritional stress led to the inhibition of the eukaryotic initiation factor 2α (eIF2α), indicating that a such stress in trypanosomatids induces a conserved translation inhibition response. However, the role of ROS in this canonical signaling pathway is still unclear.

Therefore, it is likely that ROS sensing may represent an important adaptation of the parasite to trigger the morphogenetic and biochemical transformations during the *T*. *cruzi* life cycle to proliferate or differentiate in the appropriate compartment.

Despite efforts to understand the interaction between *T*. *cruzi* and triatomines, the influence on the proliferation and/or metacyclogenesis of some abundant molecules present in distinct compartments of the vector midgut, such as: (i) heme, a byproduct of Hb digestion; (ii) hemozoin, a heme aggregate abundant in the triatomine feces [8, 21]; and (iii) urate, an important antioxidant rich in the urine of these insects [22], are still poorly studied. Here, we investigated the roles of different molecules, which are all abundant in the insect vector but with distinct redox status, in the proliferation and differentiation of *T*. *cruzi in vitro* and *in vivo*. The data presented indicate that the induction of a reductive redox environment by the addition of antioxidants, mimicking the vector rectal environment, impairs epimastigote proliferation *in vitro* and stimulates *T*. *cruzi* metacyclogenesis.

## Material and Methods

### Ethics Statement

All animal care and experimental protocols were conducted following the guidelines of the institutional care and use committee (Committee for Evaluation of Animal Use for Research from the Federal University of Rio de Janeiro, CAUAP-UFRJ) and the NIH Guide for the Care and Use of Laboratory Animals (ISBN 0–309–05377–3). The protocols were approved by CAUAP-UFRJ under registries #IBQM001 and #IBQM011. Technicians dedicated to the animal facility at the Institute of Medical Biochemistry (UFRJ) carried out all aspects related to rabbit husbandry under strict guidelines to insure careful and consistent handling of the animals.

### Parasites

*Trypanosoma cruzi* Dm28c (CT-IOC-010) was provided by the Trypanosomatid Collection of the Oswaldo Cruz Institute, Fiocruz, Brazil. Parasites were grown at 28°C for 7 days in brain-heart infusion medium (BHI, BD Bacto, USA) supplemented with 30 μM heme (Frontier Scientific, Utah, USA) and 10% fetal calf serum (FCS, Vitrocell, Campinas, Brazil), in cell culture flasks with growth area of 25 cm^2^. Parasite growth was monitored by cell counting in a Neubauer chamber.

### Epimastigote *in vitro* proliferation assays

Epimastigotes (2.5 x10^6^ parasites/mL) were grown at 28°C for 10 or 12 days in BHI medium supplemented with 10% FCS in the absence or presence of 30 μM heme, 39 μM Paraquat, 30 μM β-hematin and different concentrations (3, 20, 30 100, 300 μM) of hydrogen peroxide (H_2_O_2_), (30 μM or 1 mM) of glutathione (GSH) or NAC (30 μM or 1 mM). Afterwards, parasite growth was monitored by cell counting in a Neubauer chamber. Three independent experiments were performed in duplicate.

### Flow cytometry analysis of the cell cycle

Epimastigotes (2.5 x10^6^ parasites/mL) were grown at 28°C in BHI medium supplemented with 10% FCS in the absence (control) or presence of 30 μM heme, 30 μM NAC or 1 mM urate. After 72h of incubation, parasites were fixed in methanol for 3 min and stained with 5 μg/mL propidium iodide and 500 μL of 100 μg/mL ribonuclease A (Sigma Chemical Co., Saint Louis, MO, USA) for 10 min in the dark. Parasite DNA content was analyzed measuring the PI fluorescence (585±15 nm) in a FACSCalibur cytometer (Becton-Dickinson, San Jose, CA, USA). Fifty thousand events were acquired in the gate previously established as that of epimastigotes. Four independent experiments were performed.

### *T*. *cruzi* metacyclogenesis kinetics *in vitro*

For *in vitro* differentiation, 7-day-old epimastigotes were harvested by centrifugation and then incubated in triatomine artificial urine (TAU) medium (190 mM NaCl, 17 mM KCl, 2 mM MgCl_2_, 2 mM CaCl_2_, 8 mM phosphate buffer pH 6.0) at a density of 5×10^8^ cells/mL in the absence or in the presence of 30 μM heme, 30 μM β-hematin, 1 mM urate, 30 μM GSH or 30 μM NAC for 2 h at 28°C. Next, epimastigotes were diluted 1:100 (5 x10^6^ cells/mL) in TAU3AAG medium (TAU supplemented with 10 mM L-proline, 50 mM L-sodium glutamate, 2 mM L-sodium aspartate and 10 mM D-glucose) and the parasites were allowed to adhere in cell culture flasks with growth area of 175 cm^2^ [19] also containing the molecules mentioned above. Culture supernatants were collected after 24, 48, 72 and 96 h of incubation and stained with Panotico according to the manufacturer’s instructions. The percentage of epimastigotes and metacyclic trypomastigotes was easily differentiated morphologically by light microscopy according to the position of the kinetoplast.

### Insects and *in vivo* infections

Experimental infections were carried out using uninfected fifth-stage nymphs of *R*. *prolixus* reared at 28°C in 80% relative humidity and fed rabbit blood every 30 days in the Federal University of Rio de Janeiro colony. Twenty insects per group were artificially fed [23] heat-inactivated rabbit blood containing 5 x10^7^ epimastigotes/mL diluted in the absence or presence of 1 mM NAC or 1 mM urate. Each infected triatomine was dissected 5 days post-infection to obtain three distinct regions of the midgut: the anterior region, the posterior region and the rectum. Samples were homogenized in phosphate buffered saline pH 7.4 (PBS) and examined by direct microscopic observation (DMO). The population density at each *T*. *cruzi* stage in the different regions of the insect gut was quantified using a Neubauer chamber and classified by morphological and motility characteristics. Three independent experiments were performed. Additionally, the presence of *T cruzi* in the midgut regions of the insects was quantified by real-time PCR.

### RNA extraction and cDNA synthesis

The intestines of five fifth-stage insects were dissected in cold saline, homogenized in Trizol (Invitrogen Corporation, CA, USA) and processed according to the manufacturer’s instructions. The precipitated RNAs were then purified using the RNeasy mini kit (Qiagen) according to the manufacturer’s instructions to remove DNA and protein contaminants. The purified RNA was then used as a template for cDNA synthesis using the High Capacity cDNA reverse transcription kit (Applied Biosystems) according to the manufacturer’s instructions.

### Real-time PCR

Real-time PCR was performed using QuantiFast SYBR Green PCR Kit (Qiagen) on a Rotor-Gene Q real time cycler. Reactions were performed with either *T*. *cruzi* 195-bp repeated DNA-specific primers TCZ1–5´-CGAGCTCTTGCCCACACGGGTGCT-3´ and TCZ2–5´-CTCCAAGCAGCGGATAGTTCAGG-3 [24] or *Rp-MIP* forward 5′-CCAGTGGT-GACAATATGT´-3′ and reverse 5′-GGTACAAACA-AATTCTACG-3′. A melting curve phase program was applied with continuous fluorescence measurement between 60°C and 95°C. Negative controls for each primer consisted of a reaction with no cDNA added. To normalize the amount of tissue analyzed in each PCR reaction, we chose the *R*. *prolixus* major intrinsic protein gene (*Rp-MIP*), which encodes an aquaporin-like protein [25], as a housekeeping gene to correct for intra-sample variations in the initial sample amount, cDNA recovery and/or sample loading. Normalization with an external standard was possible because the amplification of *T*. *cruzi* and *RpMIP* sequences occurred with the same efficiency. Delta Ct values for each experimental group were averaged and relative expression was represented as 2^-ΔCt^.

### Statistical analyses

Statistical analysis was conducted with GraphPad Prism 3 software (GraphPad Software, Inc., San Diego, CA). Data are presented as the mean ± standard deviation (SD) or standard error (SE), and all experiments were repeated at least three times. Data were analyzed by one-way analysis of variance (ANOVA), and differences between groups were assessed with Tukey’s post-test. The level of significance was set at *p <* 0.05.

## Results

### Molecules with antagonistic redox status modulate epimastigote proliferation

**Fig 1:**
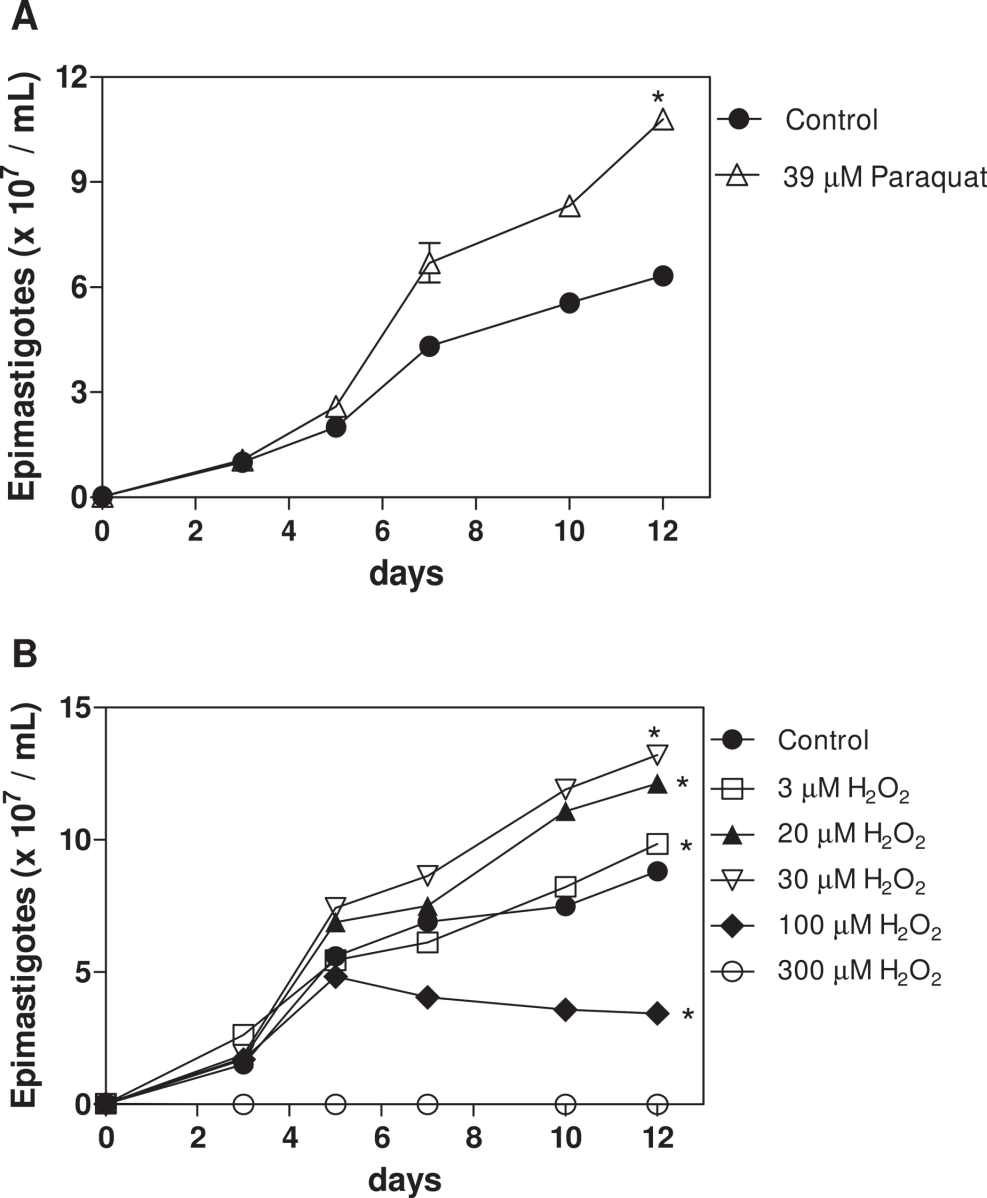
Effects of pro-oxidants on epimastigote proliferation *in vitro*. *T*. *cruzi* epimastigotes (2.5 x 10^6^cells/mL) were incubated in BHI medium supplemented with 10% FCS in the absence (control) or in the presence of **(A)** 39 μM Paraquat; **(B)** different concentrations of H_2_O_2_ (3, 20, 30, 100 or 300 μM). The graphs are representative of two independent experiments performed in duplicate of the 12^th^ day of treatment. All data are presented as the means ± standard deviation. Statistical analysis was performed for the 12^th^ day of treatment, ^*^p<0.05 compared with the control group by one-way ANOVA and Tukey’s test.

**Fig 2:**
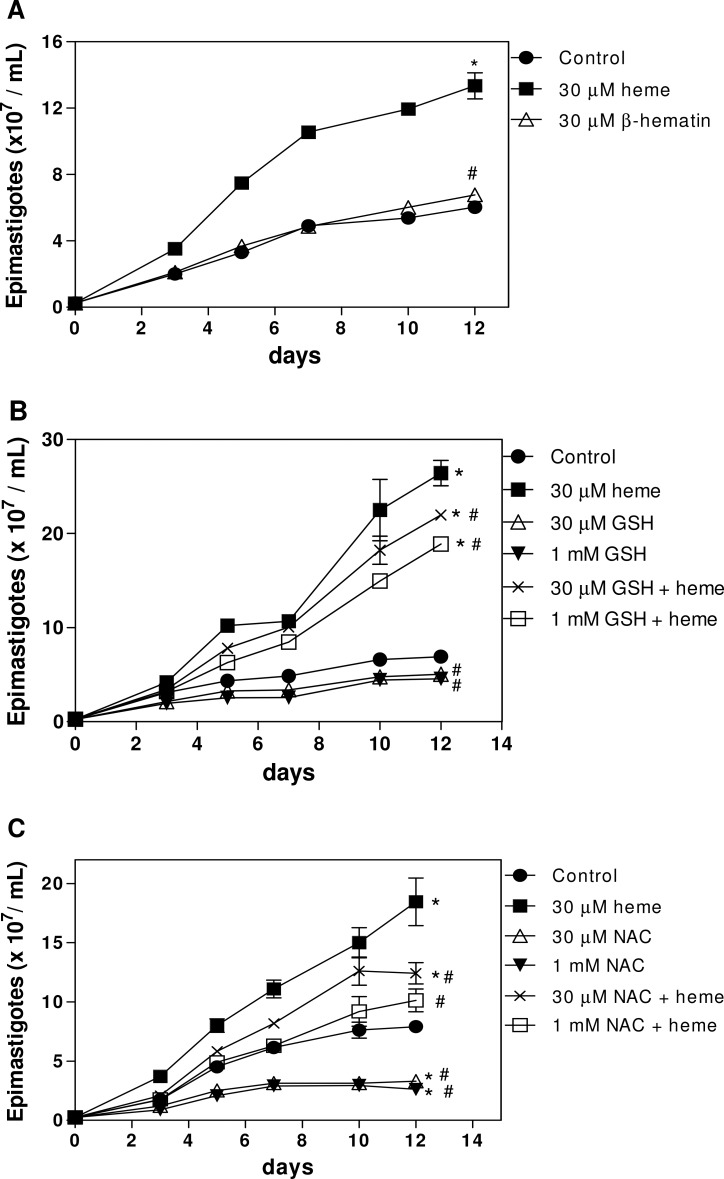
Effects of molecules of distinct redox status on epimastigote proliferation *in vitro*. *T*. *cruzi* epimastigotes (2.5 x 10^6^cells/mL) were incubated in BHI medium supplemented with 10% FCS in the absence (control) or in the presence of 30 μM heme, **(A)** 30 μM β-hematin; **(B)** different concentrations of GSH (30 μM or 1 mM) in the absence or presence of 30 μM heme; or with **(C)** different concentrations of NAC (30 μM or 1 mM) in the absence or presence of 30 μM heme. All data are presented as the means ± standard deviation. Statistical analysis was performed for the 12^th^ day of treatment, ^*^p<0.05 compared with the control group and ^#^p<0.05 compared with heme treatment by one-way ANOVA and Tukey’s test.

Our group has demonstrated the beneficial effects of heme on *T*. *cruzi* proliferation *in vitro* [9, 10]. In order to evaluate the effect of a pro-oxidant milieu upon epimastigotes growth, we challenged the parasites with two classical oxidants: H_2_O_2_ (Fig. 1A) and the well-known superoxide generator, Paraquat (Fig. 1B). As we can see in Fig. 1, both treatments increased epimastigote forms proliferation when compared to control groups (except for higher hydrogen peroxide concentrations), showing that these parasites can thrive in an oxidizing environment. We also evaluated the effect of physiological molecules present in different compartments of the invertebrate vector on epimastigote growth. Considering its abundance in the midgut of the vector, we tested the effects of β-hematin, a crystal composed of heme dimers [26], on epimastigote growth. The addition of 30 μM β-hematin to epimastigote culture in BHI medium for 12 days did not produce the same increase in proliferation induced by heme (Fig. 2A). The proliferation induced by β-hematin was similar to that of the control without heme, suggesting that the substitutions present in the porphyrin ring are required to be unbound for epimastigote proliferation *in vitro*. In a previous work, we observed that urate (1 mM) decreased epimastigote growth when compared with controls grown without heme as well as with parasites grown with heme. This inhibitory effect was partially reversed by co-incubation with 30 μM heme, suggesting a competition between these molecules of antagonistic redox status [11]. The inhibition of epimastigote proliferation by an antioxidant led us to investigate whether this effect also occurred in the presence of other reductive molecules. Therefore, we incubated parasites with GSH, a thiol-based antioxidant found in the hemolymph of triatomines. In Fig. 2B, we show that different concentrations of GSH (30 μM and 1 mM) also impaired epimastigote proliferation *in vitro* when compared with the control without heme as well as with cells grown with heme. The amino acid cysteine is responsible for the reductive power of GSH and is a classical antioxidant [27]. The incubation of epimastigotes in the presence of different concentrations of NAC (30 μM and 1 mM) for 12 days strongly inhibited cell growth compared with the control without heme and with parasites grown with heme (Fig. 2C).

### Effects of heme, NAC and urate on the cell cycle of *T*. *cruzi* epimastigotes

**Fig 3:**
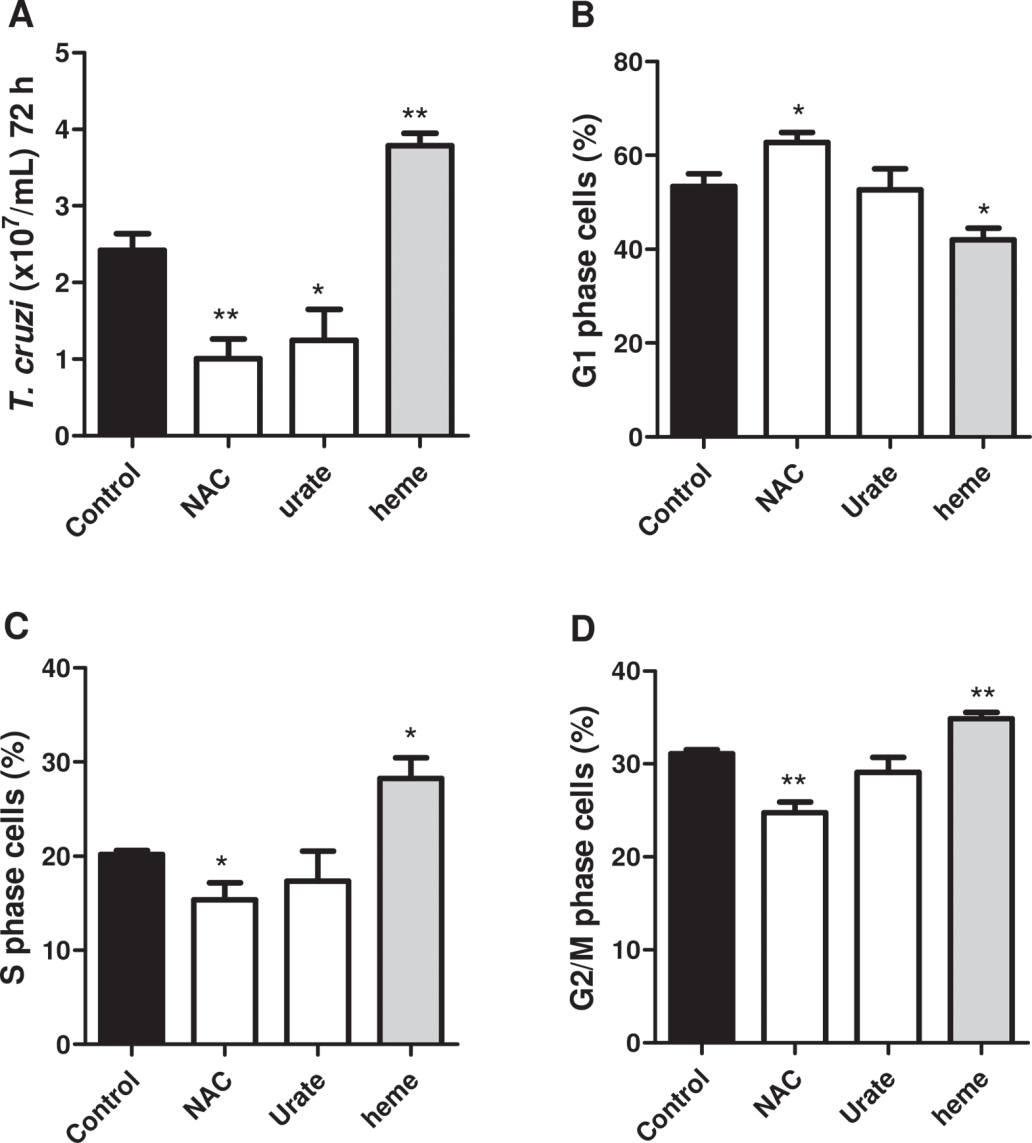
Effect of heme, NAC and urate on the epimastigote cell cycle. **(A)** Epimastigote proliferation (counting); and cell cycle analysis (flow cytometry). The graphs represent de percentage of parasite DNA in each cell cycle phase according to the treatments. **(B)** G1, **(C)** S or **(D)** G2/M phases. Cells were incubated in BHI medium in the absence (control) or presence of 30 μM NAC, 1 mM urate or 30 μM heme for 72h, and processed as described in the Material and Methods. Hypodiploid nuclei and debris were discarded from the analysis. Data represent means ± standard errors of four independent experiments, ^*^p<0.05 and ^**^p<0.001 compared with the control group by one-way ANOVA and Tukey’s test.

The effects of heme, NAC and urate on the cell cycle of *T*. *cruzi* epimastigotes is shown in Fig. 3. Treatment with heme significantly increased (by 56%) cell proliferation (3.78 x 10^7^ ± 0.36 in contrast to 2.4 x10^7^ ± 0.57 for control parasites), whereas NAC (1.005 x 10^7^ ± 0.44) and urate (1.25 x 10^7^± 0.70) significantly inhibited it (by 59% and 48%, respectively), as shown in Fig. 3A. The results showing whether these molecules alter cell proliferation at specific phases of the cell cycle are shown in Fig. 3B, 3C and 3D. Heme stimulated cell proliferation, reducing the number of cells in G1 phase (by 21.4%) (Fig. 3B) and increasing it in S phase (by 40%) (Fig. 3C) and G2/M (by 10%) (Fig. 3D), compared with the control. NAC inhibited epimastigote proliferation, arresting cells in G1 phase (by 17%) (Fig. 3B) and reducing the numbers in S phase (by 23%) (Fig. 3C) and G2/M (by 20%) (Fig. 3D). Conversely, urate inhibited cell proliferation (Fig. 3A), proportionally reducing the cell numbers in both phases of the cell cycle under the given conditions.

### Antioxidants favor the establishment of metacyclic forms of *T*. *cruzi*

**Fig 4:**
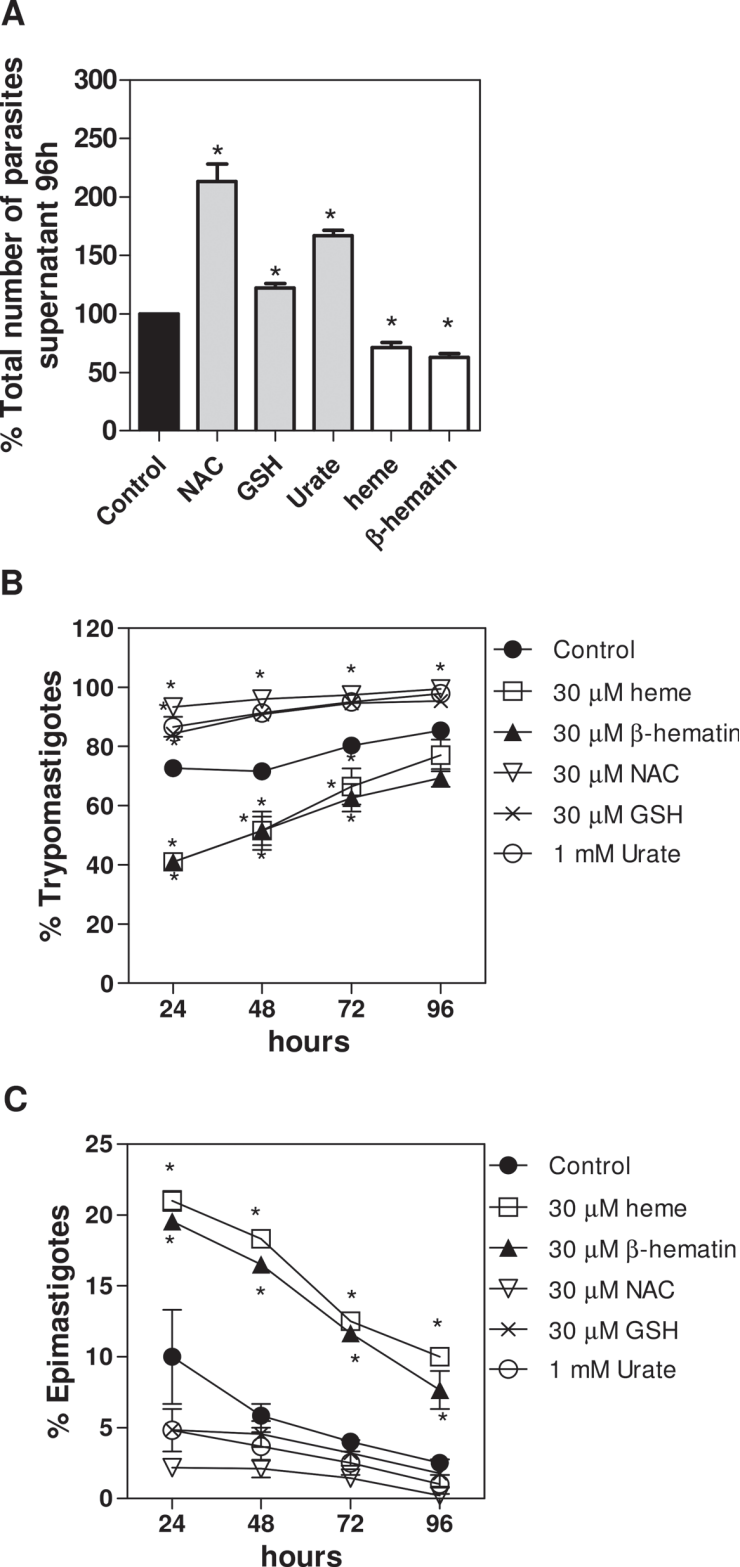
The effect of molecules of distinct redox status upon metacyclogenesis *in vitro*. *T*. *cruzi* epimastigotes were incubated in TAU3AAG medium containing 30 μM GSH, 30 μM NAC,1 mM urate, 30 μM heme or 30 μM β-hematin, as described in the Materials and Methods. **(A)** Culture supernatants were collected at different time periods, and the percentage of total parasites in the supernatant after 96 h treatment was calculated. The parasite evolutive forms were determined by light microscopy according to the kinetoplast position. **(B)** The data represent means ± standard errors of the percentage of trypomastigotes from five independent experiments. **(C)** The data represent means ± standard errors of the percentage of epimastigotes from three independent experiments, ^*^p<0.05 compared with the control group by one-way ANOVA and Tukey’s test.

To further investigate the role of molecules from the vector in the physiological process of *T*. *cruzi* differentiation, we tested the effect of heme, β-hematin, urate and the antioxidants GSH and NAC on metacyclogenesis *in vitro*. Fig. 4A shows that after the induction of metacyclogenesis, the percentage increase in total parasites/mL was approximately 67% for urate, 22% for GSH and 113% for NAC. Conversely, heme and β-hematin decreased the number of parasites/mL in the supernatant of metacyclogenesis by approximately 29% and 37%, respectively (Fig. 4A). By analyzing the percentage of trypomastigotes and epimastigotes using the position of the kinetoplast, we found an increase in trypomastigote forms (Fig. 4B) and a decrease in epimastigotes (Fig. 4C) for the parasites incubated with urate, GSH or NAC compared with the untreated group (control). The intermediate forms were also counted in the total number of parasites but are not represented graphically. Interestingly, when the cells were treated with NAC, almost 100% of the parasites in the supernatant were trypomastigote forms (Fig. 4B). Although GSH did not produce a large increase in the number of parasites/mL in the metacyclogenesis supernatant (Fig. 4A), more than 97% of the parasites were trypomastigote forms (Fig. 4B).

Moreover, epimastigotes treated with heme or β-hematin showed the opposite effect, a decrease in the percentage of trypomastigotes and an increase in epimastigote forms, compared with the control group (Fig. 4). This result is consistent with the diminished number of parasites in the supernatant of the metacyclogenesis medium.

### The antioxidants NAC and urate modulate *T*. *cruzi* metacyclogenesis *in vivo*

**Fig 5:**
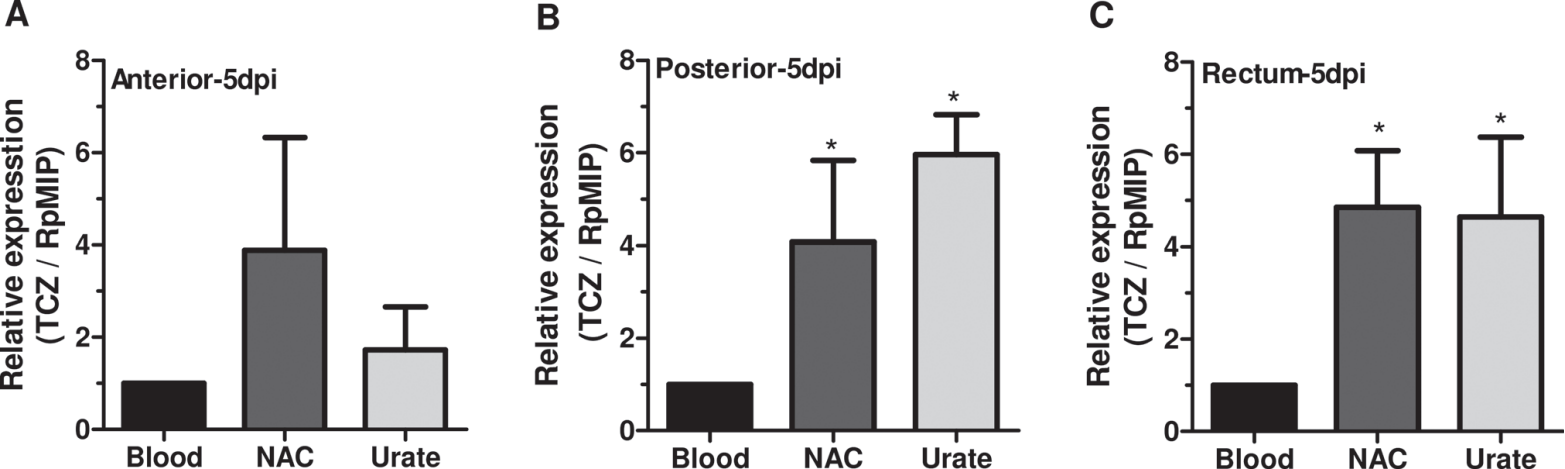
Real-time PCR analysis of parasite loads during R. prolixus infection: the effect of antioxidants in vivo 5 dpi. Fifth instar *R*. *prolixus* nymphs were fed serum-inactivated rabbit blood or blood supplemented with 1 mM NAC or 1 mM urate and 5 x 10^7^ epimastigotes/mL (at least ten insects per group in each experiment). Five days post infection, the bugs were dissected, and the total RNA of the **(A)** anterior midgut, **(B)** posterior midgut or **(C)** the rectum was extracted in TRIZOL reagent. A cDNA strand was synthetized and used as a template for amplification with TCZ primers. RpMIP was used as an endogenous control, ^*^p<0.05 compared with the blood group by one-way ANOVA and Tukey’s test.

To quantify the presence of *T*. *cruzi* in distinct regions of the insect vector digestive tract, we used primers for TCZ to amplify a 188 bp region based on the 195 bp repetitive sequences, which are expressed in all *T*. *cruzi* evolutive forms [24]. A primer for the major intrinsic protein, aquaporin-like RpMIP [25], was used to perform the relative amplification of the qPCR. Fig. 5 shows real-time PCR performed with cDNAs obtained from the different regions of the digestive tract of *R*. *prolixus* 5 days after infection with *T*. *cruzi*, and we observed that TCZ was expressed in the three regions of the insect midgut. Additionally, we evaluated whether the anti-proliferation and pro-differentiation effects of the antioxidants urate and NAC *in vitro* would also occur *in vivo*. Indeed, the presence of NAC increased TCZ expression in all three regions studied by approximately six-fold compared with the blood-fed insects. Urate also increased TCZ expression by two-fold in the anterior region of the midgut and by six-fold in the posterior region and the rectum of the triatomine (Fig. 5).

**Fig 6:**
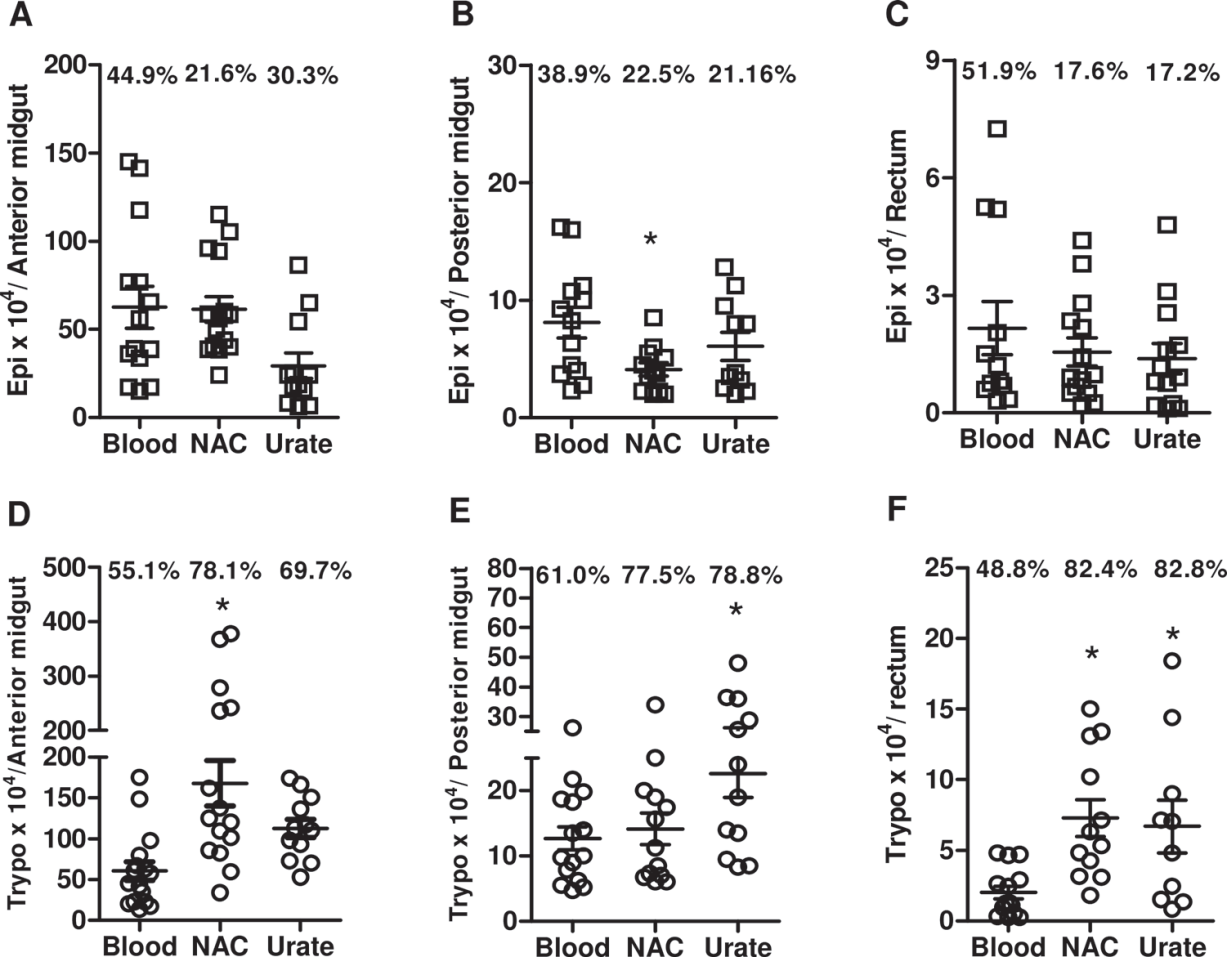
*R*. *prolixus* infection by *T*. *cruzi*: the influence of antioxidants *in vivo* 5 dpi. Fifth instar *R*. *prolixus* nymphs were fed serum-inactivated rabbit blood or blood supplemented with 1 mM NAC or 1 mM urate and 5 x 10^7^ epimastigotes/mL (at least ten insects per group in each experiment). Five days post infection, the bugs were dissected, and the parasite evolutive forms were differentiated and quantified using a Neubauer chamber in anterior midgut (A and D), posterior midgut (B and E) and in the rectum (C and F). The graphs represent means ± standard errors of at least three experiments performed in sextuplicate, ^*^p<0.05 compared with the blood group by one-way ANOVA and Tukey’s test.

Real-time PCR technology is a modern tool used to assist in studies involving the course of *T*. *cruzi* infection. However, due to its high sensitivity, it is common to encounter a higher profile of infection in qPCR analysis compared to cell counting [28]. Thus, we performed differential counts in order to elucidate and distinguish the *T*. *cruzi* developmental forms (epimastigotes or trypomastigotes) responsible for the increase in TCZ expression in the insect midgut. Five days post infection, the insects fed with the antioxidants showed a higher concentration of trypomastigotes compared with epimastigote forms in all treatments and compartments of the midgut (Fig. 6). In addition, the concentration of trypomastigote forms was significantly higher in the rectum (82.4%) of insects fed with NAC (Fig. 6F). Fig. 6E and 6F show that the blood meal supplemented with urate enriched the number of trypomastigotes in the posterior region (78.8%) and in the rectum (82.8%) of the insects. Remarkably, the treatment of vectors with NAC led to a decrease in the epimastigote forms in the posterior region of the gut (22.5%) (Fig. 6B). Using the same methodological approach, we also evaluated the expression of TCZ 11 days after infection of the insects, and we again observed an increase in expression in all regions studied, however, we did not observed a significant difference between epimastigote or trypomastigote concentrations among antioxidant treatments compared with the blood-fed group in any midgut regions (data not shown).

These results suggest that the supplementation of the blood meal with antioxidants caused a shift in the redox status of the gut compartments, increasing differentiation of the parasites in an unusual midgut region and greatly favored metacyclogenesis in the bug rectum. Notably, contrary to proliferation, the differentiation process appears to be favored by reductive environments.

## Discussion

The trypanosomatid *T*. *cruzi* presents a very complex life cycle that involves different morphological and functional stages; therefore, the adaptation to environmental changes and diverse physicochemical conditions represents an important survival mechanism. Moreover, the co-evolution between parasites and their insect vectors promoted an elegant strategy to the development of the protozoa and its maintenance in the invertebrate vector.

The heme molecule is an example of this intimate relationship between parasite and vector because it is capable of promoting the proliferation of epimastigote forms [9–11; 29], and despite its pro-oxidant role, which is potentially deleterious to cells [6], parasites present higher proliferative rates in other oxidative medium supplemented with hydrogen peroxide or Paraquat. Once more, we show that epimastigote forms appear to be well adapted to a seemingly deleterious environment Otherwise, the low molecular-weight antioxidants are known for their role in protecting against oxidative damage. Urate is thought to be one of the most important antioxidants in human plasma, acting both by forming inactive complexes with transition metals and by intercepting hydroxyl radical and organic hydroperoxides [30].

In 1931, Wigglesworth demonstrated the presence of uric acid in the Malpighi tubules and in the urine of *R*. *prolixus*. Furthermore, Graça-Souza and collaborators [7] observed that the hemolymph of *Rhodinius* prolixus contained approximately ten times more urate than human plasma. Additionally, supplementing the diet of infected mosquitoes with urate decreased the nitration of blood proteins and increased the infection by *Plasmodium berghei* in those insects [31].

By analyzing the effect of heme and reductive molecules on the proliferation of epimastigote forms, we observed that the proliferation triggered by heme, was accompanied by an increased number of cells in S phase and G2/M, supporting the stimulatory effects of heme on proliferation. In addition, a pattern of decreased parasitic growth compared with the group treated with heme was observed after treatment with reductive molecules such as NAC and urate. The role of ROS on epimastigote cell proliferation has previously been reported [11], but the present work shows, for the first time, that the inhibition of this cell proliferation by NAC resulted from cell cycle arrest in G1 phase, as observed with different mammal cells [32, 33], whereas urate did not inhibit a specific phase of the cell cycle.

Together, these data support the hypothesis of modulation between physiological molecules of antagonistic redox status, indicating an inhibitory role of reductive molecules on epimastigote proliferation and confirming the requirement of an oxidant signal, physiologically provided by heme, to promote the growth of these parasites.

It has long been known that *T*. *cruzi* differentiation is affected by factors such as pH, metabolic stress, AMPc, adenylyl cyclase by a α^D^-globin fragment present in the urine and the midgut of the insect vector [34, 35]. Additionally, after the blood meal there is an increase in trypomastigote forms in the urinary tract of the vector *Triatoma infestans* [36]. These findings support our hypothesis that urinary flux, rich in urate [22], a potent antioxidant, participates in the transformation of epimastigote forms into metacyclic trypomastigotes during the life cycle of *T*. *cruzi* [36]. Indeed, the redox state appears to contribute to the differentiation of some cell types, such as the human colon epithelial cells Caco-2 and human renal epithelial cells HEK [37, 38].

The interaction between *T*. *cruzi* and the insect vector begins with the arrival of infected blood to the midgut of the triatomine. After entering the digestive tract, the parasites find the components and products of blood digestion present in the anterior and posterior midgut of the insect. Theoretically, all of these molecules, for example, globin-derived peptides [35], hemolytic factors, lectins, hemoglobin fragments [39], enzymatic components present in the midgut of the insect [40], the free fatty acid, oleic acid [41], the gut microbiota [40, 42], the molting hormone ecdysone [43] and heme [10], are able to modulate the proliferation and differentiation of *T*. *cruzi*. Additionally, the exogenous, triterpenoid azadirachtin affects the development of *T*. *cruzi* inside the triatomine, decreasing metacyclogenesis of the parasite [44].

Despite these data and due to the complexity of the mechanisms involved in this interaction, the molecule(s) and or factor(s) necessary and sufficient to trigger the process of proliferation and differentiation of *T*. *cruzi* have not yet been identified. The biochemical interactions between *T*. *cruzi* and triatomine vectors have been investigated since 1909 [1]. Notably, the role of the abundant physiological molecules and the contrasting redox status in the *T*. *cruzi*-vector relationship has been poorly addressed.

Therefore, physiological molecules may represent a stress factor due to their high concentrations in different compartments. In fact, an increase in the density of total parasites/mL has been observed when metacyclogenesis was stimulated in the presence of urate or NAC. However, differentiation was impaired by heme or β-hematin. These effects may be related to the distinct redox character of these molecules, thus presenting contrasting roles in parasite biology.

Indeed, studies using the protozoa *Plasmodium* showed the involvement of xanthurenic acid, a reductive molecule, produced by the mosquito during the exflagellation of the parasite. Xanthurenic acid leads to gametogenesis, a process essential to malaria transmission [45, 46]. Recently, Lima and collaborators (2012) indicated that xanthurenic acid acts as an antioxidant in the yellow fever vector *Aedes aegypti* [47]. Therefore, our data are the first to demonstrate the redox status in the modulation of parasite biology.

To evaluate whether our observations *in vitro* also occurred *in vivo*, we performed quantitative analyses of parasite loads in different compartments of *R*. *prolixus* infected with *T*. *cruzi* five days post infection. Notably, corroborating the *in vitro* data, the *in vivo* infections were higher in the presence of urate and NAC, as shown by the increase in TCZ expression in all three digestive tract regions analyzed. More importantly, this increase was due to the increase in trypomastigote forms, highlighting the support of metacyclogenesis *in vivo* by a reductive environment provided by the antioxidants urate and NAC.

Recently, proteomic and functional analyses have suggested the up-regulation of proteins of the *T*. *cruzi* antioxidant network, such as Fe-superoxide dismutase A, tripanothione synthase, triparedoxin peroxidase and others in metacyclic trypomastigote compared to the epimastigote form [48–51]. In this regard, the changes in the parasite redox metabolism during metacyclogenesis could be affected by the different redox environments of each compartment of the insect. Thus, one could hypothesize that the modulation of metacyclogenesis by the antioxidants would occur as a consequence of the intensification of the parasite antioxidative defense.

Taken together, this body of work strongly suggests that, contrary to proliferation, which needs an oxidative environment to occur, differentiation occurs in more reductive surroundings. In this scenario, oxidant molecules promote proliferation, and in contrast, antioxidants appear to promote metacyclogenesis.

In a physiological context, urate represents a significant molecule both to the parasite and to the vector for the modulation of metacyclogenesis and consequent transmission of *T*. *cruzi* to its vertebrate hosts. Therefore, the assessment and definition of the contribution of the redox status in the *T*. *cruzi*-triatomine interactions toward virulence and persistence could further define it as a relevant target for the development of new pharmacological strategies that treat Chagas disease.
